# Facts, uncertainties, and opportunities in wheat molecular improvement

**DOI:** 10.1038/s41437-024-00721-1

**Published:** 2024-09-05

**Authors:** Fariba Rafiei, Jochum Wiersma, Steve Scofield, Cankui Zhang, Houshang Alizadeh, Mohsen Mohammadi

**Affiliations:** 1 Department of Agronomy, Purdue University, West Lafayette, IN USA; 2 Department of Agronomy and Plant Genetics, University of Minnesota, Northwest Research and Outreach Center, Crookston, MN USA; 3 USDA-ARS, Crop Production and Pest Control Research Unit, West Lafayette, IN USA; 4 Department of Agronomy & Plant Breeding, College of Agricultural and Natural Resource, University of Tehran, Karaj, Iran

**Keywords:** Molecular engineering in plants, Plant breeding

## Abstract

The year 2020 was a landmark year for wheat. The wheat HB4 event harboring a drought-resistant gene from sunflowers, received regulatory approval and was grown commercially in Argentina, with approval for food and feed in other countries. This, indeed, is many years after the adoption of genetic modifications in other crops. The lack of consumer acceptance and resulting trade barriers halted the commercialization of the earliest events and had a chilling effect on, especially, private Research & Development (R&D) investments. As regulations for modern breeding technologies such as genome-edited cultivars are being discussed and/or adopted across the globe, we would like to propose a framework to ensure that wheat is not left behind a second time as the potential benefits far outweigh the perceived risks. In this paper, after a review of the technical challenges wheat faces with the generation of trans- and cis-genic wheat varieties, we discuss some of the factors that could help demystify the risk/reward equation and thereby the consumer’s reluctance or acceptance of these techniques for future wheat improvement. The advent of next-generation sequencing is shedding light on natural gene transfer between species and the number of perturbations other accepted techniques like mutagenesis create. The transition from classic breeding techniques and embracing transgenic, cisgenic, and genome editing approaches feels inevitable for wheat improvement if we are to develop climate-resilient wheat varieties to feed a growing world population.

## Introduction

The year 2020 was a landmark year for wheat. The first transgenic wheat variety received regulatory approval and was grown commercially in Argentina. Bioceres Crop Solutions (Rosario, Santa Fe, Argentina) developed and brought a wheat variety to market. This event, labeled HB4, includes a drought-resistant gene from sunflowers and was developed using the microparticle bombardment method. Australia, Brazil, Columbia, New Zealand, Nigeria, and the United States have provided regulatory approval for the HB4 event for both food and feed usage directly or after processing (https://www.isaaa.org), though it cannot be grown for commercial purposes yet in the US. Our rationale for this review was to provide an overview of the vast amount of time and effort that has been spent on developing transgenic wheat that ultimately all failed to reach the marketplace prior to the approval of event HB4. While perhaps technically more challenging than other major commodities, wheat was never given the opportunity to reap benefits from this emerging technology. Lack of consumer acceptance and resulting trade barriers halted commercialization of the earliest events and had a chilling effect on, especially, private R&D investments. As regulations for cis-genic technologies are being discussed and/or adopted across the globe, we would like to propose a framework of sorts to ensure that wheat is not left behind a second time as the potential benefits far outweigh the perceived risks. As the HB4 event is a significant landmark in wheat’s cultivation history, we aim to present a perspective on the opportunities and challenges of genome-edited (GE) wheat. We outline three critical areas that center around the technology itself and perceived risks in the adoption of technology: (1) the history of wheat transformation, and the hurdles for wheat to benefit from GE technology, (2) showcase the missed opportunities in wheat with examples in model genotypes, and (3) compare the mutation loads caused by natural, chemical, or physical mutations with those caused by genome editing technology and present some established data for the analysis of relative risks associated with each method.

### A brief history of transgenic wheat and technologies

Plant breeding, or the directed evolution of a plant species to fit human needs, is as old as species domestication. While pure line selection had been practiced for (Lupton 1987; Shaltouki Rizi and Mohammadi 2023) much of the 19th century, modern plant breeding, founded on the principles of Mendelian genetics started at the turn of the 20th century. Wheat benefited from pure line selection and modern plant breeding early on. Leading names like Vilmorin in France, Biffen in England, Rimpau in Germany, Broekema in the Netherlands, Nilsson-Ehle in Sweden, Saunders in Canada, and Farrer in Australia all developed and released new wheat varieties (Lupton 1987). Starting in the 1950s with Ernie Sears’ work at the University of Missouri, wheat again played a pioneering role in chromosome engineering using wide crosses. Wheat producers today still benefit from these DNA introgressions from other species (Sears 1956). The pace of innovations in plant breeding has accelerated exponentially since the late 1980s. The ability to identify, isolate, and migrate DNA sequences with known functions, from the same or other species, assisted by molecular tools, allowed for this second revolution to occur. Transgenic varieties of crop species, commonly referred to as genetically modified organisms (GMOs), not only fundamentally changed agriculture but also changed plant breeding as a discipline and industry. This time, wheat breeders did not benefit from those same innovations that have been deployed by corn, cotton, or soybeans breeders. The two main reasons for this disparity are the technological barriers to the development of transgenic traits in wheat and societal barriers to the adoption of transgenic traits in wheat prior to the first commercialization attempts.

Rice was the first cereal to be transformed, followed by maize. There are also reports of gene transfer in wheat, rye, and barley as early as in the 1980s (Ishida et al. 2018). The first successful wheat genetic modification event was developed at the University of Florida by using the biolistic bombardment method in wheat cultivars ‘Pavon’ and ‘RH770019’ (Vasil et al. 1992). While at the beginning particle bombardment seemed revolutionary in the field of cereal transformation (Altpeter et al. 2005), a major shortfall was the variation reported in the stability, integration, and expression of the transgene (Kohli et al. 1999). In 1997 scientists at Monsanto transformed wheat by using an Agrobacterium-mediated transformation method (Cheng et al. 1997). Despite these early successes, the development of genetic engineering methods for wheat has lagged behind other important crops such as maize and rice because wheat is notoriously recalcitrant to in-vitro regeneration, and transformation in wheat is highly genotype-dependent (Shrawat and Armstrong, 2018). Based on the methodological variations historically used for wheat transformation, the methods are presented here in two broad categories based on whether or not tissue culture procedures are used.

### Transformation methods relying on tissue culture

*Agrobacterium* (Ag)-mediated methods integrate fewer copies of transgenes into the genome with better stability of transgene expression (Shrawat and Armstrong 2018). Regeneration potential of explants and stability of transgenic integration are key determinants of success when using Agrobacterium (Ag)-mediated transformation. These factors are influenced by the explant tissue, explant age, explant source, tissue culture response, and composition of culture medium, the physiological stage of the donor plant, and the interaction of these main effects (Boyko et al. 2009). Explants in tissue culture methods could include plant cell suspensions, protoplasts, plant organ cultures, meristem cultures, and pollen cultures (Kumar and Loh 2012). Plant tissue culture methodologies vary in complexity in all plant species and sometimes require long and complex processes that can lead to unpredictable and undesired modifications in the function and structure of the plant genome (Thorpe 2007). Methods that rely on tissue culture are inherently laborious and expensive, and regenerated seedlings may be less viable and more prone to diseases in the glasshouse environment. These complexities could be due to the activation of secondary metabolites in plants, which in turn, affects the growth of the new cells or explants (Thorpe 2007; Sharma and Kathayat 2021).

Methods relying on tissue culture frequently used mature or immature wheat embryos as explants. Among several types of explants (pollen, anthers, immature and mature embryos, floral organs, and the young spikes and leaves), the immature embryos seemed to be the best explants (Li et al. 2012; Hayta et al. 2019), and the most responsive to regenerate embryogenic callus in wheat (Haliloglu 2002). However, the use of mature embryos could save time and lower the workload (Yang et al. 2015). Filippov et al. (2006) demonstrated that mature embryos had more differentiated tissues and older cells than immature embryos; hence, using a high concentration of auxin seems to be essential for re-differentiation. Transformation of mature embryos shows low efficiency. When Wang et al. transformed mature embryos of wheat using a longitudinally cut, they observed transformation efficiencies (TEs) of 0.89%, 0.67%, and 0.06%, for wheat varieties including, ‘Lunxuan 208’, ‘Yumai66’, and CVs. ‘Bobwhite’, respectively (Wang et al. 2009). Ishida et al. (2015) showed that centrifugation pretreatment of immature embryos before Ag infection was effective for gene transmission. Although Ishida et al. (2015) claimed an average efficiency between 50 and 60% and a peak efficiency of 90%, other research groups were unable to repeat the claimed efficiencies in their laboratories (Hayta et al. 2019; Kuruwita Bandaralage 2023; Biswal et al. 2023). Hensel et al. (2017) demonstrated that preculture and temperature pretreatment of immature embryo cells of the spring wheat genotype ‘Bobwhite SH98-26’ may lead to reproducible production of transgenic plants with a TE of up to 15%. In their study, the strain of Agrobacterium AGL1 harboring the pGH215 plasmid was used to transfer the *hygromycin* resistance gene, under the control of an enhanced 35S promoter. This T-DNA also included a synthetic GFP with S65T mutation that was controlled by the maize ubiquitin (Ubi-1) promoter to facilitate the assessment of different treatments (Hensel et al. 2017). Mechanisms of these pretreatments are not known and it only seems they tend to render cells of maize, wheat, and rice more competent for Ag-mediated transformation (Hiei et al. 2006; Ishida et al. 2015).

Agrobacterium strains and binary vectors have a large influence on the success of transformation. Ag-mediated approaches use specific strains of Ag with chromosomal and plasmid sequences that enable the binding and transmission of the DNA into the host genome. Monocotyledonous species are not natural hosts of Ag (Potrykus 1990). Only Ag strains LBA4404 (Ach5), C58 and AGL have been reported to be successful in monocot transformation (Hellens et al. 2000b), but these have been applied with a wide range of binary vectors. Ag strains used for wheat transformation have either low or standard virulence (vir) expression, like C58C1 and LBA4404 (Khanna and Daggard 2003; Zhang et al. 2012), or are super virulent, like AGL (Lazo et al. 1991). High levels of vir gene expression appear to increase the efficiency and expand the ability of transformation on more wheat genotypes such as ‘Bobwhite’, ‘Yumai66’ and ‘Lunxuan 208’ (Wang et al. 2009).

In addition, the combination of highly competent Agrobacterium strain with effective plasmid constructions results in increased success rates for wheat transformation (Komari 1990; Bińka et al. 2012). Engineered strains such as AGL0 and AGL1 have been designed to harbor hypervirulent Ti plasmid, pTiBo542, which contains extra vir genes (Lazo et al. 1991). The hypervirulent strain AGL0 harboring pTiBo542 (Lazo et al. 1991), was reported to be superior to other strains for wheat transformation (Weir et al. 2001). The pSOUP helper plasmid harboring a DNA fragment (15 Kbp) of pTiBo542 with extra vir genes increased the transformation efficiency of T-DNA integration in wheat (Lazo et al. 1991; Hellens et al. 2000a; Wu et al. 2003). LBA4404 with the super binary pHK21 vector that included additional copies of vir genes B, C, and G from pTiBo542 has also been reported to be successful in wheat transformation. In this research, immature embryo‐derived calli of spring wheat cv. ‘Veery #5’ were transformed by LBA4404 harboring either pHK22 binary vector or pHK21 super binary vector, the latter harboring an additional set of the *vir* genes. The results showed that binary vectors with high vir expression led to the improvement of transformation frequency in cereals However, it should be noted that LBA4404 harboring helper plasmids with standard vir gene expression have also been successful in generation transformants in the cultivar ‘Bobwhite’ (Hu et al. 2003; Khanna and Daggard 2003).

The success of tissue culture-based methods is sensitive to the composition of culture medium and is also highly dependent on genotypes (Haliloglu 2014). For example, He et al. (1988) demonstrated that white callus transformation frequency is enhanced by increasing the concentration from half-strength to full or double-strength of macro elements. Haliloglu (2014) reported that for a callus initiation medium, the concentration of 2,4-D and the developmental stage of the immature embryo played critical roles in the success of somatic embryos. In addition, the concentration and composition of the plant growth regulators (PGRs) have tremendous effects on the success of tissue culture in wheat. While the results of many assays showed that the most common type of auxin used in wheat and other cereals was 2,4-D (Filippov et al. 2006), a few studies indicated that dicamba was more efficient than 2,4-D and other auxin types in non-endosperm and endosperm-based investigations in wheat (Mendoza and Kaeppler 2002; Filippov et al. 2006). Papenfuss and Carman (1987) showed that because dicamba was easily metabolized, shoot regeneration increased. Wang et al. (2022) indicated that overexpression of the wheat gene TaWOX5 from the WUSCHEL family significantly enhanced the transformation efficiency with less genotype dependency compared to other approaches. The successful transformation of 31 common wheat cultivars led to the creation of transgenic plants using TaWOX5. Aadel et al. (2018) observed using acetosyringone (200 μM), resulted in TEs of 0.66% and 1.00% for the genotypes ‘Rajae’ and ‘Amal’, respectively. However, it is well known, that the impact of genotype is even greater than that of medium composition (Mathias and Simpson 1986). Over decades, in methods that relied on tissue culture, wheat has been shown to be a recalcitrant species to in-vitro regeneration, completely genotype-dependent (Hu et al. 2019), and with low transformation efficiency.

### Transformation methods not relying on tissue culture

Methods that do not rely on tissue culture, known as *in-planta* transformation, use Ag co-cultivation or biolistic bombardment approaches primarily and transform cells of the sexual organs of the plant or seed (Chee and Slightom 1995; Keshavareddy et al. 2018). In biolistic approaches, a relatively large-scale DNA segment, sometimes more than 100 kb, can integrate into the genome (Zhang et al. 2012). Partier et al. (2017) reported producing transformants of ‘Bobwhite’, showing expression in the T1 and T2 generations, by using a biolistic method with a linear dephosphorylated 53 kb cassette consisting of a 44 Kb segment from an Arabidopsis gene linked to a selectable marker and reporter genes. Other *in-planta* methods techniques such as co-cultivation of plant tissue with Ag, sometimes with vacuum infiltration, infecting germinating seeds, and floral dipping have been described in the literature (Bent 2000). These methods are much less time-consuming and less labor-intensive than plant tissue culture (Xu et al. 2008). The floral dip method pioneered in Arabidopsis is the most commonly used method to date. With this method plants are transformed by dipping the flower buds into an Ag suspension (Clough and Bent 1998). The floral dip has also been developed and optimized for cereals including rice (1.4% TE) (Ratanasut et al. 2017), maize (3.3% TE) (Mu et al. 2012), and wheat (0.44% TE) (Zale et al. 2009). It has also been reported that transgenic wheat can be produced by an *in-planta* method where Ag is directly injected into the apical meristem wheat cultivar ‘Shiranekomugi’. This was observed to result in 33% transformation when measured by PCR analysis (Supartana et al. 2006). Because in-planta approaches bypass callus culture and do not need regeneration, they are being pursued to develop genotype-independent protocols for plant species that are recalcitrant to regeneration. *In-planta* methods using immature embryos as explants have been developed for Ag (Tamás et al. 2001) and biolistic-mediated (Hamada et al. 2017) transformation in wheat. Most transgenic plants with Ag-mediated methods will have a single copy of the transgene (Tassy et al. 2014; Yao et al. 2006).

## Moving forward with transgenic approaches in wheat

Several innovations have resulted in breakthroughs for overcoming genotype dependency and increasing TE. The genetic background seems to be the main factor in plant regeneration (Kausch et al. 2019). Based on the strong correlation observed between chromatin accessibility and gene expression, a transcriptional regulatory network (TRN) driving callus induction after auxin treatment was established, and a total of 446 transcription factors (TFs) were identified. These studies demonstrated that the sequential expression of genes mediating cell fate transition during regeneration was induced by auxin in coordination with changes in chromatin accessibility, H3K27me3, and H3K4me3 status. Furthermore, TaDOF5.6 and TaDOF3.4 can significantly improve the transformation efficiency of different wheat varieties. Thus, the data provides molecular regulatory insights for the wheat shoot regeneration process and potential novel targets for improving transformation efficiency in wheat (Liu et al. 2023).

Similar to any other processes in plants, it is logical to assume that different wheat genotypes have different gene expression patterns in a given explant organ, and therefore respond differently to the same regeneration condition, and therefore, are genetically different in ability to regenerate and be transformed. Some genes are key in developmental regulation and could break genotype dependency, thereby improving regeneration efficiency (Altpeter et al. 2016). Expression of these developmental regulators, often referred to as morphogenes or booster genes, can reprogram the somatic cells to the embryonic cells (Gao 2021). Adjustments for the expression levels of these booster genes were suggested as potential breakthroughs for overcoming genotype dependency.

These adjustments were reported as BBM-WUS (Baby boom and Wuschel) system in maize and wheat (Zhou et al. 2022), and LEC1/LEC2 systems in Arabidopsis (Boulard et al. 2017; Stone et al. 2001) and more recently, the GRF-GIF systems Arabidopsis, rice, maize, triticale and wheat (Kim 2019; Liebsch and Palatnik 2020) were developed. For example, WUS is involved in activating cell division and avoiding stem cells prematurely differentiating into shoot apical meristem (SAM) cells (Wójcik et al. 2020). The WUS protein moves in upper cell layers where stem cells are located and induces cell division while impeding stem cell differentiation (Tvorogova et al. 2019). Increases in the expression of WUS resulted in the spontaneous development of somatic embryo cells on root explants of Arabidopsis (Zuo et al. 2002). In Arabidopsis hypocotyls, mutations with reduced expression of WUS mutant abrogated regeneration of the shoot in-vitro, while overexpression led to the regeneration of shoots on a hormone-free medium (T. Q. Zhang et al. 2017). WUS overexpression has resulted in increased responses of embryogenic cells in other plants (Solís-Ramos et al. 2009; Zheng et al. 2014). BBM encodes an AINTEGUMENTA-LIKE (AIL) APETALA2/ethylene-responsive factor (AP2/ERF) that is preferably expressed in developing embryo and seed cells and can activate a cascade that induces embryo development from differentiated plant cells (Boutilier et al. 2002; Horstman et al. 2017). Overexpression of a pair of morphogenic booster genes, WUS2 and BBM has improved transformation and regeneration efficiency in recalcitrant genotypes and species like sorghum, inbred lines of sugarcane, maize, rice (Lowe et al. 2018; Mookkan et al. 2017). A dwarf phenotype was reported when using morphogenic genes in wheat and excision-based strategies i.e., removing the BBM-WUS to recover a normal plant phenotype (Gordon-Kamm et al. 2019).

The delivery of two morphogenic regulator genes, maize Zm-Baby Boom (ZmBbm) and Zm-Wuschel2 (ZmWus2) for Agrobacterium-mediated transformation of wheat, results in a significant boost in transformation efficiency ranging from 58% to 75% (Johnson et al. 2023). The LEC TFs (e.g., LEC1, LEC2, and FUS3) are principal regulators in the maturation and embryogenesis of Arabidopsis (Braybrook et al. 2006). LECs launch the cellular environment which enhances the development of seed embryo cells in maturation and morphogenesis of zygotic embryogenesis (ZE) (Braybrook et al. 2006; Harada 2001). Loss-of-function mutants of LEC1 and LEC2 showed pleiotropic impacts on embryo development, particularly in the regulation of late embryogenesis. The mutations resulted in the loss of embryo organ identity, defective storage, desiccation intolerance, reserve accumulation, and premature post-germinative growth (Harada 2001). When LEC1 and LEC2 are ectopically overexpressed, they induce the development of embryos in vegetative cells, activating the formation of embryo-like structures and expression of embryo-specific RNAs (Stone et al. 2001). This outcome highlights the critical role of LECs genes in somatic embryogenesis (SE). Their expression is limited to seed development, as well as ZE and SE (Boulard et al. 2017). Expression of GROWTH_REGULATING factors (GRFs) has improved wheat TEs (Hu et al. 2021; Kim 2019; Liebsch and Palatnik 2020). Every GRF protein interacts with the associated coactivator, called GRF-INTERACTING FACTOR (GIF), to make a functional complex (GRF-GIF) (Kim 2019; Shimano et al. 2018). A new study showed that a morphogenic gene, GRF4 with its corresponding coactivator GRF-INTERACTING FACTOR 1 (GIF1) boosted wheat transformation (Debernardi et al. 2020; Qiu et al. 2022). Previous studies indicated that transient expression of developmental regulators TaGRF4 and TaGIF1 forming the booster complex (TaGRF4-TaGIF1), could enhance genome editing frequency and the efficiency of regeneration in wheat (Qiu et al. 2022). The BBM, LEC1, and LEC2 TFs have been shown to be the main regulators of the totipotency control in plant cells, as ectopic overexpression of either TF initiates somatic embryo formation in Arabidopsis seedlings without stress treatments or exogenous growth regulators (Horstman et al. 2017).

## Moving away from transgenic mutations may increase public acceptance

A clear definition of different genetic modification routes helps a lot in de-risking, solving social challenges, and improving consumer and market acceptance. Genetically modified (GM), or transgenic organisms, as per the World Health Organization, are referred to those genetic material changes in microorganisms, plants, or animal species that do not occur in natural recombination or mating (Kumar et al. 2020; Zhang et al. 2016). In contrast, the term cis-genic was coined by Schouten et al. (2006) referring to modifications in a recipient plant by a naturally derived gene from a cross-compatible species that includes introns, native promoters, and terminators in the normal sense orientation. An example of this is the introgression and expression of the high molecular weight glutenin subunit 1Dy10 of durum wheat, which was sourced from bread wheat (Gadaleta et al. 2008). The final product should not have any kind of foreign DNA or selectable markers. The cis-genic route is what can be achieved via natural outcrossing and selection and therefore, should bear fewer social concerns than transgenics. The genome (or gene) editing (GE) route enables precise changes to the specific sites on the genome – e.g., small changes such as single nucleotide insertion or deletion from a gene. The gene editing technology uses the clustered regularly interspaced short palindromic repeat (CRISPR)/CRISPR-associated protein (CRISPR/Cas) (Rajput et al. 2021), which may provide effective tools for crop improvement. For example, knockouts of the three homeologous Mildew Resistance Locus (MLO) genes TaMLO-A1, TaMLO-B1, and TaMLO-D1 resulted in powdery mildew resistance in wheat (Acevedo‐Garcia et al. 2017). These tools were used in a multiplex manner by using the tRNA-gRNA technique to simultaneously target three genes TaMLO, TaLpx-1, and TaGW2 genes (Wang et al. 2018). Besides initial knockout applications, newer techniques were developed to increase or decrease levels of gene expressions by CRISPR activator (CRISPRa) and CRISPR interference (CRISPRi) (Zhou et al. 2022). In 2013, three independent teams applied CRISPR/Cas9 for use in tobacco, wheat, rice, and Arabidopsis (Li et al. 2013; Nekrasov et al. 2013; Shan et al. 2013). Since then, rapid enhancements in the CRISPR/Cas systems, like CRISPR/Cpf1 with applications such as base editing (Zetsche et al. 2015) and the nucleotide substitution tools for base editing, have established gene editing as a widely adopted, easy-to-use targeted genetic modification tool that has been applied to numerous crops (Shimatani et al. 2017; Zong et al. 2017).

Genome editing has the potential to open a new door for the development of wheat varieties with superior phenotypes and permit their commercialization even in countries where GM crops are not accepted or permitted. Adding desired traits and eliminating those that are unwanted from elite varieties can remarkably enhance overall crop yield and tolerance to environmental stresses and/or improve end-use quality. A single-guide RNA (sgRNA) directs the Cas9 endonuclease to a precise locus in the genome with a complementary sequence. There it hybridizes and creates double-strand breaks (DSBs), thereby generating short insertion or deletion (indels), potentially with a desired phenotypic outcome. The applications can include base editing (Chen et al. 2019; Li et al. 2021), transgene-free genome editing by transient expression (Zhang et al. 2016), large chromosomal deletions, and resolution of locus structure and mutation efficiency (Arora and Narula 2017). Evidence also shows that viral vectors may also be used to express CRISPR/Cas9. For example, the barley stripe mosaic virus-based gRNA delivery vector in a transgenic wheat line harboring Cas9 could edit a targeted locus without a T-DNA expressing the gRNA remaining in the wheat genome (Hu et al. 2017). In-vitro methods can accelerate tool development for variations in Cas protein codon modifications or optimizations, the promoters used for sgRNA, sgRNA target-sites, Cas9 proteins, and also various vector designs (Čermák et al. 2017; Endo et al. 2019).

Wheat can be improved for grower traits and consumer traits. Important examples of input traits are increases in yield and disease resistance. Some of the traits that were developed by using knockout strategies are listed in Table 1. Examples of input traits are grain weight, resistance to diseases such as powdery mildew and Fusarium head blight disease, and herbicide resistance (Table 1). Genome editing has also been shown to be effective in developing breeding-facilitating traits such as male sterility or increases in haploid induction rate. Examples of output traits are changes in nutritional values or improving health-related traits such as high amylose content and lower gluten flour. The most obvious bottleneck shown by this table is that the majority of these manipulations were only made in two outdated varieties, neither of which is currently commercially grown anywhere in the world.

**Table 1:** Examples of trait developments via genome editing in wheat.

Target gene	Trait	Nucleases	Edit	Wheat genotype	Reference
*TaGW2* ^ax^	Grain weight	cas9	Knockout	Kenong 199, YZ814, Bobwhite	(Liang et al. 2017; Wang et al. 2018)
*TaGASR7* ^b^	Grain length	nCas9 (H840A)	Prime editing	Kenong 199	(K. Wang et al. 2018a)
*aEDR1* ^cx^	Powdery mildew	Cas9	Knockout	Kenong 199	(Y. Zhang et al. 2017)
*TaMLO-A1* ^ax^	Powdery mildew	Cas9	Knockout	Bobwhite & Kenong 199	(Wang et al. 2014)
*TaHRC* ^ax^	*Fusarium graminearum*	Cas9	knockout	Bobwhite	(Su et al. 2019)
*TaLpx-1* ^ax^	*Fusarium graminearum*	Cas9	knockout	Bobwhite	(Wang et al. 2018)
*TaNFXL1* ^*bz*^	*Fusarium graminearum*	Cas9	knockout	Fielder	(Cui et al. 2019)
*EPSPS* ^bw^	Herbicide resistance	Cas9	Knockout	Fielder	(Arndell et al. 2019)
*TaPDS* ^ay^	Carotenoid biosynthesis pathway	Cas9, dCas9	Knockout, base editing	Fielder	(Howells et al. 2018)
*TaLOX2* ^ax^	Seed longevity and viability	nCas9-D10A/dCas9	base editing	Bobwhite	(Zong et al. 2017)
*TaSBEIIa* ^ax^	High amylose content	Cas9	knockout	Bobwhite & Zhengmai 7698	(Li et al. 2021)
*TaMs1* ^ax^	Male sterility	Cas9	knockout	Fielder & Gladius	(Tucker et al. 2017)
*TaCENH3a* ^ax^	High haploid induction rate	Cas9	knockout	Fielder	(Lv et al. 2020)
*TaZIP4* ^ay^	Increased crossover frequency	Cas9	knockout	Fielder	(Rey et al. 2018)
*α-gliadin* ^bw^	Low-gluten	Cas9	knockout	Bobwhite	(Sánchez-León et al. 2018)

Explant: ‘a’ immature explant, and ‘b’ protoplast transfection; Method: ‘x’, Biolistic transformation, ‘y’, agrobacterium, ‘z’ Microprojectile bombardment, ‘w’ Protoplast transformation.

To advance genetic gains, manipulation of multiple traits per unit of time is required. Molecular strategies such as multiplex genome editing (Wang et al., 2018b) by the simultaneous expression and transfer of multiple sgRNAs has been demonstrated. Traditionally, targeting multiple genes requires constructing multiple sgRNA expression constructs (containing a sgRNA, a Pol III promoter, and a terminator), which can be carried by single T-DNA, however, this approach faces problems such as limitation in restriction sites, large-size T-DNAs, and reductions in editing efficiency.

Approaches have been developed to simplify multi-target genome editing in cereals. A few examples of these approaches are listed in Table 1, including the use of tRNA-processing enzymes and Csy-type ribonuclease 4 (Csy4) to simultaneously express multiple gRNAs, for manipulating genes TaEPSP, TaMLO, and TaUbi (Table 1). It may also be possible to manipulate traits by engineering increases or decreases in the level of gene expressions which can benefit quality and nutritional traits. The inactivation of the catalytical site by a dead mutation in Cas9 (dCas9) has been re-programmed to regulate the target gene at the transcriptional level. The dCas9 does not cleave DNA strands. It only forms a DNA identification complex with sgRNA in order to be recruited to the complementary DNA double-strand in the promoter region. The presence of this complex blocks the identification of RNA polymerase activity and numerous transcriptional binding factors, thereby interfering with transcriptional initiation and elongation sterically. Thus, the production of target gene transcripts is completely blocked (Piatek et al. 2015; Qi et al. 2013). Other modifications have been developed for transcriptionally modifying levels of gene expressions. For example, to increase or decrease gene expression the transcriptional activation domains (TADs) or transcriptional repression domains (TRDs) can be fused to dCas9 C-terminus in order to down-regulate or upregulate the gene expression effectively (Heyl et al. 2008; Zhou et al. 2022).

### Facts, myths, perceived risks, and barriers to adoption of modern molecular breeding techniques

Besides technological limitations, social barriers have also slowed wheat biotechnology. In 1996 Monsanto approached the wheat breeding programs at South Dakota State University, North Dakota State University, Montana State University, the University of Minnesota, and Agriculture and Agri Food Canada in western Canada to start developing glyphosate-tolerant wheat (Wiersma, personal communications). Event 33391, conferring glyphosate tolerance to wheat, had been created in cv. ‘Bobwhite’ (Zhou et al. 1995). It was field tested across 14 locations in 1999 and 2000 and 13 locations in 2000. Zhou et al. (2003) showed that the transgenic event with or without glyphosate application yielded as high as the non-transgenic Bobwhite. Eventually, event 33391 was backcrossed into elite wheat spring germplasm (Mergoum et al. 2005). Monsanto submitted two provisional patent applications (No. 60/236,653 and No. 60/236,762) on September 29, 2000. This was followed by the formal application on May 23, 2002 (Pub. No. US 2002/0062503). Two years later Monsanto announced that the development and efforts to bring glyphosate-resistant spring wheat to market in the US and Canada would be halted indefinitely (No-Till Farmer Magazine 2004). The drivers of the decisions to enter and withdraw from the wheat domain were not driven by the technical challenges as detailed above, but rather by the various stakeholder groups. While it was individual producers and grower organizations that initially wanted wheat to gain the same (perceived) benefits already imparted to corn, soybeans, and cotton, it was the lack of consumer acceptance in the EU and Japan that eventually sealed its fate.

Already in 1998, merely two years after the first transgenic crops had entered the US marketplace, researchers started to recognize that consumer perception of risks and benefits associated with individual events and the resulting transgenic crops determined its acceptance (Frewer et al. 1998). Philip Dale (1999) (Dale n.d.) argued in his commentary that the EU’s reluctance towards transgenic crops was in part because of the rather different relationship EU consumers have with food in comparison to US consumers. Ultimately, the lack of market acceptance for the event in the EU and Japan doomed the efforts to bring glyphosate-resistant spring wheat to the field as the risk/reward equation for growers and the US food industry was no longer favorable.

One possible way to lower the perceived risk is to compare the mutation landscape that is created by nature and accepted breeding methods to modern biotechnology methods including trans- and cis-genic transformation and genome editing. Natural mutations during DNA replication and meiosis produce significantly higher mutations than any targeted induced mutation over time. DNA replication occurs countless numbers of times. Depending on the genome size of the organism, it has to copy and transmit the exact same sequence of nucleotides to the daughter cells. Though DNA replicates with very high fidelity, errors are inevitable. Most of these mistakes are corrected through various DNA repair processes. However, some of these errors are not and thus become permanent mutations. When these mutations occur in cells that give rise to gametes, they can even be transmitted to subsequent generations. In some cases, replication errors could involve insertions or deletions of nucleotide bases due to strand slippage or strand loops, resulting in the addition or deletion of nucleotides. In the experimental model organism, *Arabidopsis thaliana*, it has been reported that the rate of nucleotide substitution is 6.95 × 10^−9^ (SE ± 2.68 × 10^−10^) and 1.30 × 10^−9^ (±1.07 × 10^−10^) per site per generation insertion/deletion (Weng et al. 2019). Applying the same rate on the wheat genome, would produce 6.95 × 10^−9^ × ~ 17 Gbp = 118 sites per generation, and 1.30 × 10^−^^9^ × ~ 17 Gbp = 22 insertion/deletion per generation. These mutations accumulate by the rate that seeds are replicated for breeding or production and result in a significant inter-varietal genetic difference.

In a pangenome study of hexaploid bread wheat across 18 cultivars (Montenegro et al. 2017) more than 36 million inter-varietal single nucleotide polymorphisms were reported. From a pangenome size of 140,500 ± 102 genes, 81,070 ± 1631 genes were considered as core genome. This data indicates a huge structural variation in wheat germplasm for gene presence-absence. The same study concluded that 12,150 gene sequences are absent in cultivar ‘Chinese Spring’ which was used to develop the wheat genome assembly (Montenegro et al. 2017).

In addition to single nucleotide changes, gene deletions, or insertion within species, nature also changes the genetic constitution of closely related species via translocations that shuffle chromosomal segments. In cereals, translocation has resulted in chromosomal rearrangements that led to intraspecific differentiation (Badaeva et al. 2007). Of nearly 500 genotypes of wheat and triticale from Europe, Asia, and USA, involving various levels of ploidy, 58 major types of chromosomal rearrangements in 139 wheat accessions (70 tetraploid and 69 hexaploid) were identified. These rearrangements were either between two A-genome chromosomes, A- and B-genome chromosomes, two B-genome chromosomes, A- and D-genome chromosomes, and B- and D-genome chromosomes, and finally, the 1B:1R translocation, which is rather common in bread wheat (Badaeva et al. 2007). In fact, hexaploid wheat cultivars, which contain a segment of the rye chromosome 1R translocated onto the long arm of the 1B chromosome of wheat (1B/1R) are widely used in agriculture (Heslop-Harrison et al. 1990). A recent multi-genome study of wheat (Walkowiak et al. 2020) revealed extensive structural rearrangements, differences in gene content, and introgressions from wild relatives in wheat.

Horizontal gene transfer or laterally acquired genes can also occur in nature. Horizontal gene transfer (HGT) can be defined as the acquisition of genetic material from another organism without being its offspring. In eukaryotes, HGT now appears to occur more frequently than originally thought. Many studies are currently detecting novel HGT events among distinct lineages using next-generation sequencing (Quispe-Huamanquispe et al. 2017) In plants, one well-studied example of HGT is the transfer of the tumor-inducing genes (T-DNAs) from some Agrobacterium species into their host plant genomes. The fifth most produced food crop, sweet potato (*Ipomoea batatas*), carries ancient T-DNA and therefore could be considered a natural GMO (Kyndt et al. 2015). This discovery is fate-changing because it is the first natural GMO crop that people have consumed for millennia without any health risk ever having been reported. Sweet potato is not the only plant with reported natural T-DNA insertion. Another important example of HGT and natural GMOs is tea. *Camellia sinensis* var. *sinensis* cv. ‘Shuchazao’ possesses a single 5.5 kb T-DNA fragment (CaTA) that is composed of three dormant T-DNA genes and an inverted repeat of 1 kb at each end (Chen et al. 2022). In 1983 a sequence with homology to T-DNA was reported in *Nicotiana glauca* (White et al. 1983) and now T-DNAs have been reported in two other species *N. otophora* and *N. tobacum*. Yellow toadflax (*Linaria vulgaris* Mill.) is another plant species belonging to the Plantaginaceae (Cal-IPC 2004) and has T-DNA reported in its genome (Matveeva and Lutova 2014). In a recent report, Raimondeau et al. (2023) reported frequent movement of DNA between individuals within the grass family without sexual reproduction, through an unknown mechanism. They identified laterally acquired genes in five genomes from Alloteropsis genus by detecting Alloteropsis genes nested in distantly related clades of grasses thanks to modern sequencing technologies.

Chemical (EMS) and physical (X-rays and gamma-rays) mutagens can induce various types of mutations that are distributed randomly throughout the genome, and are useful in both genetic research and breeding. Examples of mutation-released wheat cultivars are ‘Carolina’ in Chile, ‘Jiaxuan’ and ‘Jienmai 2’ in China, and ‘Tatara’ in Pakistan (www-pub.iaea.org). The frequency and types of mutations are a direct result of the dosage and rate of exposure to the mutagen (Zhang and Hao 2020). We only have started to recognize the substantial genetic mutation loads as a result of chemical or physical mutagens after the advent of modern sequencing techniques. For example, gamma irradiation in rice included large deletions (9.4–129.7 kbp) among other small (1–16 bp) deletions and single-base substitutions (Morita et al. 2009). Li et al. (2019) characterized the mutations induced by Gamma Ray in rice by whole genome sequencing. They found that on average, 5.9 insertions and 17.7 deletions along with 57.0 single-base substitutions were observed in mutant lines at M_5_ generation. In addition, they identified 0.6 structural variations on average in the forms of large deletions or insertions, inversions, duplications, and reciprocal translocations in each gamma-ray-irradiated mutant. Despite these drastic changes, food derived from mutation breeding varieties is widely used and accepted and was never demonized by consumers. In mutation-derived varieties unexpected, unintended, and uncontrollable nature of non-targeted mutations were never a concern prior to public release. Despite facing public acceptance issues, there are many transgenic events that have already been approved. As of July 28, 2023, the number of transgenic events for major crops that were approved across the globe include wheat (2), alfalfa (5), rice (8), tomato (11), soybean (43), potato (51), cotton (67), and corn (244). Examples of transgenic events that are already approved in the USA are listed in Table 2.

**Table 2:** List of GMO traits in important world food and industrial crops.

Crop and event name	ISAAA code	Trade name	GM trait
Wheat - *Triticum aestivum*			
HB4 Wheat	IND-ØØ412-7	HB4 Wheat	Drought stress tolerance
MON71800	MON-718ØØ-3	Roundup Ready™ wheat	Glyphosate herbicide tolerance
Rice - *Oryza sativa L*.			
GR2E	IR-00GR2E-5	Golden Rice	Mannose metabolism, Enhanced Provitamin A Content
LLRICE601	BCS-OSØØ3-7	Liberty Link™ rice	Glufosinate herbicide tolerance
Potato - Solanum tuberosum L.			
E12	SPS-ØØE12-8	Innate® Cultivate	Lowered Free Asparagine, Reduced Black Spot, Lowered Reducing Sugars
SEMT15-02	NMK-89935-9	Shepody NewLeaf™ Y potato	Coleopteran insect resistance, Viral disease resistance, Antibiotic resistance
Tomato - *Lycopersicon esculentum*			
FLAVR SAVR™	CGN-89564-2	FLAVR SAVR™	Antibiotic resistance, Delayed fruit softening
Apple - Malus x Domestica			
GD743	OKA-NBØØ1-8	Arctic™ “Golden Delicious” Apple	Antibiotic resistance, Non-Browning
GS784	OKA-NBØØ2-9	Arctic™	Antibiotic resistance, Non-Browning
NF872	OKA-NBØØ3-1	Arctic™ Fuji Apple	Antibiotic resistance, Non-Browning
Pineapple - Ananas comosus			
EF2-114	FDP-ØØ114-5	Rosé	Delayed ripening/senescence, Modified fruit color

### Conclusion and outlook

Technical challenges and consumer acceptance have put wheat at a disadvantage compared to corn and soybeans. Gene editing approaches using CRISPR technologies may allow the seed industry to overcome the consumer’s reluctance towards transgenic wheat. Genome editing can add beneficial traits directly to finished cultivars that are widely grown at a much faster pace than traditional trait integration, thereby increasing genetic gains. In other words, future cultivar development could be a combination of traditional plant breeding by developing adapted and high-yielding varieties and then finishing them with the addition of desired traits via editing.

The reasons that put wheat at a historic disadvantage for the use of genetic modifications are public sensitivity and technological bottlenecks in efficiently transforming wheat. While highly efficient and genotype-independent wheat transformation protocols are still lacking, even with the presence of working protocols, wheat editing would still be disfavored when compared to the more “acceptable” crop improvement approaches such as mutation breeding, despite the very large number of background mutations that occur during those processes, or when compared to naturally GM foods such as sweet potato. Public disapproval seems to be a short-sighted issue for food grains such as wheat.

There are plenty of examples over the course of human history in which disruptive technologies have been adopted at lightning speed and changed whole industries or facets of society almost overnight. Acceptance of these disruptive technologies by society at large, and by extension elected officials, is often much slower. Hybrid corn and semi-dwarf stature rice and wheat gave rise to the Green Revolution, yielded tremendous increases in global food, feed, and fiber security, and saved millions from starvation. The logical next step is not limiting the benefits of gene editing approaches to only corn, cotton, or soybeans in select markets but extending its potential to all crops, including wheat, that mankind relies on for food security. While the consumers and producers are weighing their options for GE wheat, earlier breeding successes with a single allele could perhaps serve as a model for global exploitation of what genome editing can offer for food security. Such a multilateral approach involving government partners across the globe having a stake in its success might be advantageous.
